# Association of *ZFHX3* Genetic Polymorphisms and Extra-Pulmonary Vein Triggers in Patients With Atrial Fibrillation Who Underwent Catheter Ablation

**DOI:** 10.3389/fphys.2021.807545

**Published:** 2022-01-05

**Authors:** Inseok Hwang, Oh-Seok Kwon, Myunghee Hong, Song-Yi Yang, Je-Wook Park, Hee Tae Yu, Tae-Hoon Kim, Jae-Sun Uhm, Boyoung Joung, Moon-Hyoung Lee, Hui-Nam Pak

**Affiliations:** Yonsei University College of Medicine, Yonsei University Health System, Seoul, South Korea

**Keywords:** atrial fibrillation, *ZFHX3*, genetic polymorphism, extra-pulmonary vein, recurrence, catheter ablation

## Abstract

**Background:** The *ZFHX3* gene (16q22) is the second most highly associated gene with atrial fibrillation (AF) and is related to inflammation and fibrosis. We hypothesized that *ZFHX3* is associated with extra-pulmonary vein (PV) triggers, left atrial (LA) structural remodeling, and poor rhythm outcomes of AF catheter ablation (AFCA).

**Methods:** We included 1,782 patients who underwent a *de novo* AFCA (73.5% male, 59.4 ± 10.8 years old, 65.9% paroxysmal AF) and genome-wide association study and divided them into discovery (*n* = 891) and replication cohorts (*n* = 891). All included patients underwent isoproterenol provocation tests and LA voltage mapping. We analyzed the *ZFHX3*, extra-PV trigger-related factors, and rhythm outcomes.

**Result:** Among 14 single-nucleotide polymorphisms (SNPs) of *ZFHX3*, rs13336412, rs61208973, rs2106259, rs12927436, and rs1858801 were associated with extra-PV triggers. In the overall patient group, extra-PV triggers were independently associated with the *ZFHX3* polygenic risk score (PRS) (OR 1.65 [1.22–2.22], *p* = 0.001, model 1) and a low LA voltage (OR 0.74 [0.56–0.97], *p* = 0.029, model 2). During 49.9 ± 40.3 months of follow-up, clinical recurrence of AF was significantly higher in patients with extra-PV triggers (Log-rank *p* < 0.001, HR 1.89 [1.49–2.39], *p* < 0.001, model 1), large LA dimensions (Log-rank *p* < 0.001, HR 1.03 [1.01–1.05], *p* = 0.002, model 2), and low LA voltages (Log-rank *p* < 0.001, HR 0.73 [0.61–0.86], *p* < 0.001, model 2) but not the *ZFHX3* PRS (Log-rank *p* = 0.819).

**Conclusion:** The extra-PV triggers had significant associations with both *ZFHX3* genetic polymorphisms and acquired LA remodeling. Although extra-PV triggers were an independent predictor of AF recurrence after AFCA, the studied AF risk SNPs intronic in *ZFHX3* were not associated with AF recurrence.

## Introduction

Catheter ablation of atrial fibrillation (AF) is the most effective rhythm control method for AF patients (Hindricks et al., 2021). Circumferential pulmonary vein isolation (CPVI) is the most important procedure during AF catheter ablation (AFCA), and the efficiency of a long-lasting CPVI is improving with the catheter technology (Peyvandi et al., 2016; Reddy et al., 2020; Pak et al., 2021). However, AF is a progressive degenerative disease and shows a constant rate of recurrence despite an effective CPVI (Park et al., 2019). In particular, the rhythm outcome of a second ablation is poorer in patients with a well-maintained *de novo* PVI, suggesting the role of extra-PV triggers in the mechanism of AF recurrence (Kim et al., 2017; Park et al., 2020). In patients with significant atrial substrate remodeling, isoproterenol provocation-induced extra-PV triggers are more common and the post-AFCA recurrence rate is higher in an extra-PV trigger group compared with a non-extra-PV trigger group, and an extra-PV trigger ablation lowers the recurrence rate (Lee et al., 2018; Kim et al., 2021). However, little is known about the mechanism and appropriate techniques for the mapping and ablation of extra-PV triggers of AF. AF is a heritable disease influenced by more than 100 genes (Roselli et al., 2018). Among them, the *ZFHX3* gene is the second most highly associated with AF and affects inflammation, fibrosis, matrix deposition, and atrial structural changes (Nojiri et al., 2004; Park et al., 2013; Liu et al., 2014; Tomomori et al., 2018; Alí et al., 2019). Therefore, in this study, we explored whether extra-PV triggers, which are found frequently in patients with acquired atrial structural remodeling, are influenced by the genetic background. In addition, since the *ZFHX3* gene can affect not only atrial remodeling, but also the reaction to catheter ablation lesions, we evaluated how it affects the AFCA outcome.

## Materials and Methods

### Study Population

**FIGURE 1:**
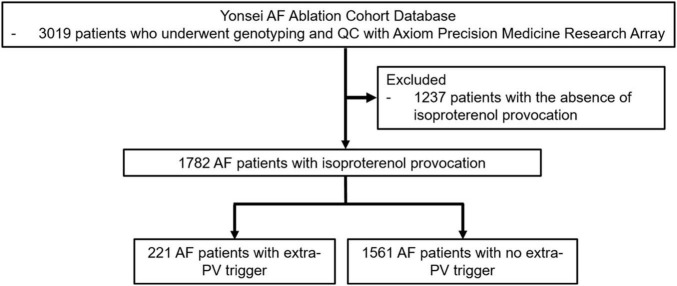
Flow chart of the enrollment and analysis of the study population. The 221 AF patients who underwent *de novo* AFCA procedures with extra-PV triggers and 1561 AF patients without extra-PV triggers were included in the study population.

This study was conducted as a single-center retrospective study. The study protocol followed the principles of the Declaration of Helsinki, and it was approved by the Institutional Review Board at Yonsei University Health System. All patients provided written consent for inclusion in the Yonsei AF Ablation Cohort prior to the study (ClinicalTrials.gov identifier: NCT02138695). A total of 1,782 patients underwent a *de novo* AF catheter ablation from March 2009 to January 2021 and post-procedural isoproterenol provocation tests (Figure 1). Among those patients, 221 had extra-PV triggers. The study exclusion criteria were (1) permanent AF refractory to electrical cardioversion, (2) AF with valvular disease grade > 2; (3) prior cardiac surgery with concomitant AF surgery, and (4) patients who did not undergo an isoproterenol provocation test or (5) genome-wide association study (GWAS).

### Genotyping and Selection of ZHFX3 Related Single Nucleotide Polymorphisms

Genomic DNA was extracted from peripheral blood samples using the QuickGene DNA whole blood kits with a QuickGene mini 80 (KURABO, Osaka, Japan). DNA genotyping data were obtained using the Axiom Precision Medicine Research Array (PMRA, Thermo Fisher Scientific, MA, United States). This DNA chip array consisted of more than 900,000 markers to assist in the translation of the research results to clinical insight.

### Echocardiographic Characteristics

Transthoracic echocardiography (Sonos 5500, Philips Medical System, Andover, MA, United States or Vivid 7, GE Vingmed Ultrasound, Horten, Norway) was used to measure the cardiac chamber size, left ventricular ejection fraction, trans-mitral Doppler flow velocity, and ratio of the early diastolic peak mitral inflow velocity to the early diastolic mitral annular velocity (E/Em). The procedure was in accordance with the American Society of Echocardiography guidelines (Nagueh et al., 2009).

### Electrophysiological Study and Atrial Fibrillation Catheter Ablation

We used the Prucka CardioLab™ Electrophysiology system (General Electric Medical Systems, Inc., Milwaukee, WI, United States) to record the intracardiac electrograms and generated 3D electroanatomical maps (NavX, Abbott, Inc., Chicago, IL, United States; CARTO system, Biosense Webster, Diamond Bar, CA, United States) using a circumferential PV-mapping catheter through a long sheath. The 3D electroanatomical maps were merged with 3D spiral CT images. We conducted transseptal punctures and obtained multi-view pulmonary venograms. Systemic anticoagulation was conducted with intravenous heparin to maintain an activated clotting time of 350–400 s during the procedure. An open-irrigated tip catheter (30–60 W; 47°C) was utilized for the AFCA. The CPVI was conducted during the *de novo* procedure with bidirectional block. Cavotricuspid isthmus block was generated for most patients during the procedure unless there was an atrioventricular conduction disease. We performed an additional empirical linear ablation including a roof line, posterior interior line (posterior box lesion), anterior line, left lateral isthmus ablation, right atrial ablation, or complex fractionated electrogram ablation at the operator’s discretion.

### Isoproterenol Provocation and Ablation Endpoint

After completing the protocol-based AF ablation procedure, AF or atrial tachycardia (AT) was induced by high-current ramp pacing (250–120 ms, 10 mA, pulse width 5 ms, Bloom Associates, Denver, CO, United States) from the high right atrial (RA) electrodes as previously described (Kim et al., 2021). We infused isoproterenol (5∼20 μg/min depending on β-blocker use with a target heart rate of 120 bpm) for at least 3 min before induction and maintained this for 3 min after the induction of AF or AT. Then, internal cardioversion was conducted utilizing a biphasic shock (2–20 J) with R wave synchronization (Lifepak12, Physiocontrol Ltd., Redmond, WA, United States). Electrical cardioversion was performed under induced deep sedation, while all the other procedures were conducted under conscious sedation. After successful electrical cardioversion, we stopped the isoproterenol infusion and waited for 10 min to detect any immediate recurrence of AF or AT originating from the extra-PV trigger. The existence of extra-PV triggers was defined by the immediate recurrence of AF or AT within the 10 min of an isoproterenol cool-down after cardioversion. If further AF triggers were observed under the isoproterenol effect, we determined the potential location of the extra-PV triggers based on the contact bipolar electrograms and conducted a quick and detailed 3D-activation mapping with a multielectrode catheter. Based on 3D mapping of the non-PV triggers, we ablated those triggers with 35–50 W for 10 s for each lesion until the elimination.

### Post-ablation Management and Follow-Up

After AFCA, the patients were discharged without taking any anti-arrhythmic drugs, with the exception of those who had recurrent extra-PV triggers, symptomatic frequent atrial premature beats, non-sustained atrial tachycardia, or an early recurrence of AF during admission. The patients visited the outpatient clinic at 1, 3, 6, and 12 months and every 6 months thereafter or whenever symptoms occurred. All patients underwent electrocardiography (ECG) at every visit. Twenty-four hour Holter monitoring was also conducted at 3 and 6 months, annually for 2–5 years, and then biannually after 5 years. Holter and event monitor recordings were obtained when patients experienced palpitations suggestive of an arrhythmia recurrence. We defined AF recurrence as any episode of AF or atrial tachycardia (AT) lasting at least 30 s in duration. Any ECG documentation of an AF recurrence within a 3-month blacking period was classified as an early recurrence, whereas AF recurrence after 3 months was diagnosed as a clinical recurrence.

### Statistical Analysis

Continuous variables were summarized as the mean ± SD and compared by either an independent Student’s *t*-test or Mann-Whitney *U*-test as appropriate. Categorical variables were summarized as the number (percentage of the total group) and compared by either a chi-square test or Fisher’s exact test as appropriate. A multivariable logistic regression analysis was used to identify the independent predictors of the existence of extra-PV triggers. A multivariable Cox regression analysis was conducted to identify the independent predictors of an AF clinical recurrence. A Kaplan-Meier analysis with a log-rank test was conducted to calculate the AF recurrence-free survival over time and AF recurrence rates according to the clinical and genetic factors. A *P*-value less than 0.05 was considered statistically significant. We constructed a polygenic risk score (PRS) using two SNPs (rs1336412 and rs61208973) and excluded three (linkage disequilibrium, *r*^2^ > 0.2). We calculated the PRS using a β-coefficient (effect size) and the number of effect alleles. The PRS was divided into quartiles for a statistical analysis performed using SPSS software (SPSS Inc., Chicago, IL, United States).

## Results

### Baseline Characteristics and *ZFHX3* Single-Nucleotide Polymorphisms Associated With Extra- Pulmonary Vein Triggers

A group of AF patients [73.5% male, 59.4 ± 10.8 years old, and 65.9% paroxysmal AF (PAF)] who received a trigger provocation was included in our study. All patients underwent a genetic analysis by a GWAS. Table 1 summarizes the characteristics of the patients with extra-PV triggers and those without. To find the extra-PV trigger-associated SNPs, we divided the included patient population into cohort 1 (73.7% male, 59.3 ± 10.8 years old, and 65.3% PAF) and cohort 2 (73.2% male, 59.4 ± 10.7 years old, and 66.4% PAF, Supplementary Table 1). Logistic regression analyses showed that 9 *ZFHX3* SNPs were associated with extra-PV triggers in cohort 1 and 10 SNPs in cohort 2 (Supplementary Table 2). Among them, the 5 SNPs, rs13336412, rs61208973, rs2106259, rs12927436, and rs1858801, were associated with extra-PV triggers (*p* < 0.05, Table 2). The 5 AF risk SNPs evaluated in this study were located in intronic regions of the *ZFHX3* gene on chromosome 16. We displayed the individual position and risk/ref allele, risk allele frequency (RAF) for replicated SNPs in Table 2. In 1,782 patients, 1,733 (97.3%) had *ZFHX3*-associated SNPs.

**TABLE 1:** Characteristics of the extra-PV triggers.

	Overall (*n* = 1,782)	Extra-PV trigger (*n* = 221)	No extra-PV trigger (*n* = 1,561)	*P*
Age, years	59.4 ± 10.8	60.6 ± 9.9	59.2 ± 10.9	0.115
Gender (male), n (%)	1,309 (73.5%)	147 (66.5%)	1,162 (74.4%)	0.015
PAF, n (%)	1,169 (65.9%)	139 (63.8%)	1,030 (66.2%)	0.478
AF Duration, months	38.8 ± 43.6	50.1 ± 50.3	36.8 ± 42.0	<0.001
BMI, kg/m^2^	24.9 ± 3.0	24.5 ± 3.022	24.9 ± 3.0	0.055
CHA2DS2-VASc Score	1.7 ± 1.5	1.8 ± 1.6	1.7 ± 1.5	0.660
**Comorbidities, *n* (%)**				
Heart failure, *n* (%)	238 (13.4%)	35 (15.8%)	203 (13.0%)	0.247
Hypertension, *n* (%)	803 (45.1%)	93 (42.1%)	710 (45.5%)	0.341
DM, *n* (%)	258 (14.5%)	28 (12.7%)	230 (14.7%)	0.414
Stroke, *n* (%)	197 (11.1%)	26 (11.8%)	171 (11.0%)	0.719
Vascular disease, *n* (%)	190 (10.7%)	13 (5.9%)	177 (11.3%)	0.014
**Echocardiographic parameters**				
LA Dimension, mm	41.3 ± 6.2	41.2 ± 6.4	41.4 ± 6.1	0.650
LAVI, ml/m^2^	37.3 ± 13.0	40.2 ± 14.1	36.9 ± 12.8	< 0.001
LVejection fraction	63.4 ± 8.1	63.5 ± 7.2	63.4 ± 8.2	0.894
E/Em	10.2 ± 4.2	10.4 ± 4.4	10.2 ± 4.2	0.659
LA Volume by CT, ml	151.1 ± 48.8	157.9 ± 50.5	150.2 ± 48.5	0.018
Pericardial fat volume, cm^3^	108.2 ± 55.3	96.3 ± 52.5	109.8 ± 55.5	0.003
Mean LA voltage, Mv	1.5 ± 0.7	1.3 ± 0.692	1.5 ± 0.7	0.004
Procedure time, minutes	168.1 ± 55.8	171.1 ± 57.7	167.7 ± 55.6	0.382
Ablation time, seconds	4261.7 ± 1975.6	4074.0 ± 2059.1	4288.3 ± 1962.7	0.122
**Ablation lesions, n (%)**				
CPVI	1,782 (100%)	221 (100%)	1,561 (100%)	NA
CTI ablation	1,610 (90.3%)	191 (86.4%)	1,419 (91.0%)	0.030
Posterior box isolation	515 (28.9%)	67 (30.3%)	448 (28.8%)	0.632
Anterior line	380 (21.3%)	54 (24.4%)	326 (20.9%)	0.228
Complications, n (%)	74 (4.1%)	15 (6.8%)	59 (3.8%)	0.036
Clinical recurrence, *n* (%)	592 (33.2%)	100 (45.2%)	492 (31.5%)	< 0.001

*PAF, Paroxysmal atrial fibrillation; DM, Diabetes mellitus; BMI, Body mass index; LA, Left atrium; LAVI, Left atrial volume index; LV, Left ventricle; E/Em, Mitral inflow velocity/mitral annulus tissue velocity; CPVI, Circumferential pulmonary vein isolation; CTI, Cavotricuspid isthmus.*

**TABLE 2:** Association of extra-PV triggers for the *ZFHX3* SNPs.

Extra-PV trigger						Cohort 1 (*n* = 891)			Cohort 2 (*n* = 891)		
SNP	Chr	Position	Risk/ref allele	RAF (%)	Region	OR	CI (95%)	*p*	OR	CI (95%)	*P*
rs13336412	16	72981949	C/A	79.1	Intron	0.74	0.55–1.00	0.049	0.64	0.48–0.86	0.003
rs61208973	16	73025093	C/T	73.3	Intron	1.39	1.04–1.87	0.028	1.35	1.02–1.78	0.035
rs2106259	16	72990459	G/T	54.0	Intron	1.46	1.08–1.97	0.014	1.37	1.02–1.84	0.034
rs12927436	16	72994943	C/A	52.4	Intron	1.39	1.03–1.88	0.032	1.45	1.09–1.92	0.010
rs1858801	16	73019560	T/C	62.3	Intron	1.38	1.02–1.87	0.037	1.52	1.15–2.00	0.003

*SNP, Single nucleotide polymorphism; Chr, Chromosome; RAF, Risk allele frequency; OR, Odds ratio; CI, Confidence interval.*

### Characteristics of Patients With Extra- Pulmonary Vein Triggers

**FIGURE 2:**
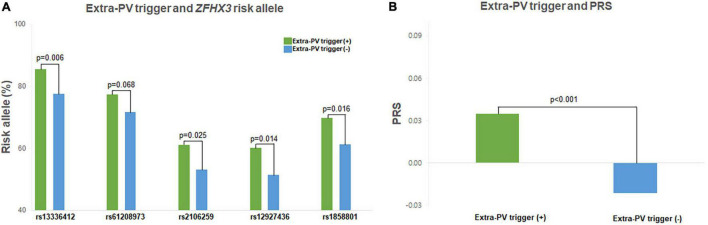
Characteristics of the extra-PV triggers. The extra-PV trigger risk allele frequency and PRS were higher in all 5 SNPs **(A)** and AF patients with extra-PV triggers **(B)**.

Table 1 compares 221 patients with extra-PV triggers and 1,561 patients without. In patients with extra-PV triggers, the AF duration was longer (*p* < 0.001) and mean left atrial (LA) voltage lower (*p* = 0.004). The risk allele frequencies of the rs13336412 (85.5 vs. 77.5%, *p* = 0.006), rs2106259 (61.1 vs. 53.1%, *p* = 0.025), rs12927436 (60.2 vs. 51.3%, *p* = 0.014), rs1858801 (69.7 vs. 61.3%, *p* = 0.016), and PRS (0.04 ± 0.12 vs. -0.03 ± 0.11, *p* < 0.001) were significantly higher in the extra-PV trigger group than in those without (Figure 2). In the multivariate logistic regression analyses, for model 1, female (OR 0.73, [0.53–1.00], *p* = 0.049), no pre-existing vascular disease (OR 0.47, [0.26–0.85], *p* = 0.013), and PRS for *ZFHX3* (odds ratio [OR] 1.65, [1.22–2.22], *p* = 0.001, Model 1) and a lower LA voltage (OR 0.74, [0.56–0.97], *p* = 0.029, Model 2) were independently associated with extra-PV triggers, but each of 5 SNPs was not independently associated with extra-PV trigger in the multivariate logistic regression analyses (Table 3). A regional specificity of extra-PV triggers was observed in the superior vena cava (12.9 vs. 29.6%, *p* = 0.039) in patients with rs13336412 (Supplementary Table 3).

**TABLE 3:** Multivariate analysis of the extra-PV triggers.

	Univariate			Multivariate (Model 1)			Multivariate (Model 2)		
Extra-PV trigger	OR	95% CI	*p*	OR	95% CI	*p*	OR	95% CI	*P*
Age	1.01	1.00–1.03	0.068	1.01	1.00–1.03	0.087	1.01	1.00–1.03	0.167
Gender (male)	0.68	0.50–0.92	0.013	0.73	0.53–1.00	0.049	0.74	0.50–1.09	0.122
PAF	0.90	0.67–1.21	0.478						
AF DURATION (*n* = 999)	1.01	1.00–1.01	0.001						
**Comorbidities**									
Heart failure	1.26	0.85–1.86	0.247						
Hypertension	0.87	0.65–1.16	0.342						
DM	0.84	0.55–1.28	0.415						
Stroke	1.08	0.70–1.68	0.719						
Vascular disease	0.49	0.27–0.87	0.016	0.47	0.26–0.85	0.013	0.41	0.19–0.87	0.019
BMI	0.95	0.91–1.00	0.055						
CHA2DS2-VASc Score	1.02	0.93–1.12	0.659						
**Echocardiographic parameters**									
LA dimension	1.00	0.97–1.02	0.650						
LA voltage (*n* = 1322)	0.68	0.52–0.88	0.004				0.74	0.56–0.97	0.029
LV ejection fraction	1.00	0.98–1.02	0.893						
E/Em	1.01	0.97–1.04	0.659						
***5 ZFHX3* SNPs**									
rs13336412	1.76	1.17–2.63	0.006	1.52	0.96–2.40	0.075	1.55	0.88–2.71	0.129
rs61208973	1.37	0.98–1.93	0.069						
rs2106259	1.39	1.04–1.85	0.026	0.97	0.62–1.51	0.882	0.92	0.54–1.59	0.773
rs12927436	1.43	1.07–1.91	0.014	1.19	0.77–1.84	0.430	1.22	0.72–2.08	0.463
rs1858801	1.45	1.07–1.97	0.016	1.24	0.90–1.72	0.196	1.13	0.76–1.68	0.558
PRS	1.64	1.22–2.21	0.001	1.65	1.22–2.22	0.001	1.42	0.97–2.08	0.070

*OR, Odds ratio; CI, Confidence interval; PAF, Paroxysmal atrial fibrillation; DM, Diabetes mellitus; BMI, Body mass index; LA, Left atrium; LV, Left ventricle; E/Em, mitral inflow velocity/mitral annulus velocity; PRS, Polygenic risk score.*

*Model 1: Age, gender, vascular disease, rs13336412, rs2106259, rs12927436, rs1858801 adjusted.*

*Model 2: Age, gender, vascular disease, LA voltage, rs13336412, rs2106259, rs12927436, rs1858801 adjusted.*

*PRS was adjusted separately from 5 ZFHX3 SNPs for multivariate analysis.*

### Factors Associated With Clinical Recurrence After Atrial Fibrillation Catheter Ablation

**FIGURE 3:**
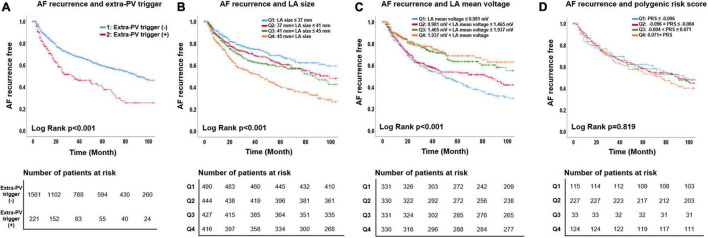
Association of AF recurrence with the extra-PV triggers, LA mean voltage, LA size, and polygenic risk score. The AF recurrence rate was higher for the extra-PV trigger **(A)**, larger LA size **(B)**, and low LA mean voltage **(C)** groups. The PRS for extra-PV triggers was not associated with AF recurrence **(D)**.

During 49.9 ± 40.3 months of follow-up, clinical recurrence was significantly higher in patients with extra-PV triggers (Log-rank *p* < 0.001, Figure 3A), a bigger LA size (Log-rank *p* < 0.001, Figure 3B), and a lower LA voltage (*n* = 1,322, Log-rank *p* < 0.001, Figure 3C). In the Cox regression analysis, extra-PV trigger (hazard ratio [HR] 1.89, [1.49–2.39], *p* < 0.001), a bigger LA size (HR 1.04, [1.02–1.05], *p* < 0.001, Model 1), and a lower LA voltage [HR 0.73, (0.61–0.86) *p* < 0.001, Model 2] were independently associated with clinical recurrence of AF after AFCA (Table 4). However, neither a Kaplan-Meier (Figure 3D) nor Cox regression (Table 4) analysis showed a relationship between the *ZFHX3* PRS and AF recurrence after catheter ablation.

**TABLE 4:** Multivariate analysis of AF recurrence.

	Univariate			Multivariate (Model 1)			Multivariate (Model 2)		
AF Recurrence	HR	95% CI	*p*	HR	95% CI	*p*	HR	95% CI	*p*
Age	1.01	1.00–1.01	0.075	1.00	0.99–1.01	0.851	1.00	0.99–1.01	0.735
Gender (Male)	0.88	0.74–1.05	0.163	0.86	0.69–1.06	0.162	1.01	0.78–1.30	0.958
PAF	0.56	0.47–0.66	<0.001	0.67	0.55–0.81	<0.001	0.78	0.62–0.97	0.026
AF duration (n = 999)	1.00	1.00–1.00	0.095						
**Comorbidities**									
Heart failure	1.52	1.22–1.91	<0.001	1.01	0.74–1.38	0.945	1.01	0.71–1.42	0.974
Hypertension	1.12	0.95–1.31	0.183						
DM	1.06	0.84–1.33	0.637						
Stroke	1.12	0.88–1.44	0.360						
Vascular disease	0.91	0.71–1.17	0.469						
BMI	1.01	0.99–1.04	0.327						
CHA2DS2-VASc Score	1.06	1.01–1.12	0.012	1.02	0.94–1.09	0.684	1.01	0.93–1.10	0.775
**Echocardiographic parameters**									
LA dimension	1.05	1.04–1.06	<0.001	1.04	1.02–1.05	<0.001	1.03	1.01–1.05	0.002
LA voltage (*n* = 1,322)	0.61	0.52–0.70	<0.001				0.73	0.61–0.86	<0.001
LVejection fraction	0.99	0.98–1.00	0.044	1.00	0.98–1.01	0.582	0.99	0.98–1.01	0.446
E/Em	1.02	1.00–1.04	0.038	0.99	0.97–1.02	0.597	0.99	0.97–1.02	0.503
Extra-PV trigger	1.94	1.56–2.40	<0.001	1.89	1.49–2.39	<0.001	2.01	1.52–2.65	<0.001
***5 ZFHX3* SNPs**									
rs13336412	1.07	0.87–1.31	0.513						
rs61208973	1.21	1.00–1.46	0.048	1.17	0.96–1.42	0.120	1.13	0.91–1.42	0.268
rs2106259	1.05	0.89–1.23	0.557						
rs12927436	1.03	0.87–1.21	0.733						
rs1858801	1.11	0.94–1.31	0.229						
PRS	1.01	0.88–1.16	0.855						

*HR, Hazard ratio; CI, Confidence interval; PAF, Paroxysmal atrial fibrillation; DM, Diabetes mellitus; BMI, Body mass index; LA, Left atrium; LV, Left ventricle; E/Em, mitral inflow velocity/mitral annulus velocity; PRS, Polygenic risk score.*

*Model 1: Age, gender, PAF, AF duration, heart failure, CHA2DS2-VASc Score, LA dimension, LV ejection fraction, E/Em, extra-PV trigger, and rs61208973 adjusted.*

*Model 2: Age, gender, PAF, AF duration, heart failure, CHA2DS2-VASc Score, LA dimension, LA voltage, LV ejection fraction, E/Em, extra-PV trigger, and rs61208973 adjusted.*

### Genetic Effects on AF Recurrence Associated With Extra- Pulmonary Vein Triggers

The post-AFCA recurrence rate was significantly higher in patients with extra-PV triggers and the *ZFHX3* risk allele than in those with extra-PV triggers and no *ZFHX3* risk allele (Log-rank *p* = 0.024) or those without extra-PV triggers (*p* < 0.001, Supplementary Figure 1A). We also compared AF recurrence between extra-PV trigger (+) *ZFHX3* (–) group and extra-PV trigger (–) group and both of groups did not have statistical difference (*p* = 0.623, Supplementary Figure 1A). In patients without extra-PV triggers, the presence of a *ZFHX3* risk allele did not affect the AF recurrence after AFCA (Log-rank *p* = 0.749, Supplementary Figure 1B).

## Discussion

### Main Findings

In this retrospective analysis of a single-center cohort study, we found an association between the extra-PV triggers and genetic variants of *ZFHX3* for the mechanism of AF among the patients who underwent AFCA. The extra-PV triggers were independently associated with both the genetic background, including the *ZFHX3*, and acquired atrial remodeling. During 49.9 ± 40.3 months of follow-up, the existence of extra-PV triggers and LA remodeling were independently associated with a clinical recurrence of AF after AFCA but not with the *ZFHX3.* Finding suggested that *ZFHX3* SNPs were associated with the extra-PV trigger, but not AF recurrence after AFCA.

### Extra-Pulmonary Vein Triggers and Outcome of Atrial Fibrillation Catheter Ablation

The extra-PV triggers are a major factor of AF recurrence after AFCA (Lee et al., 2018; Kim et al., 2021). Extra-PV triggers are associated with a low atrial voltage, being female, lower body mass index, absence of hypertension, and ventricular diastolic dysfunction in patients with paroxysmal AF (Kawai et al., 2019; Watanabe et al., 2021). Kim et al. (2021) recently reported that the existence of extra-PV triggers in repeat ablation procedures was related to women, diabetes, previous empirical extra-PV ablation, and cardiac parasympathetic nerve activity. Although an extra-PV trigger ablation mapped after an isoproterenol provocation significantly lowered the AF recurrence, it was still worse than that in patients without extra-PV triggers, even after an extra-PV trigger ablation (Kim et al., 2016; Lee et al., 2018).

### Roles of *ZFHX3* in Atrial Fibrillation

The *ZFHX3*, located on chromosome 16q22, is significantly associated with the mechanism of AF (Gudbjartsson et al., 2009) and is the second most highly AF associated genome in the multiethnic GWAS consortium (Roselli et al., 2018). The function of *ZFHX3* is related to the myogenic and neuronal differentiation by regulating the signal transducers and activators of transcription 3 (STAT3) (Nojiri et al., 2004; Liu et al., 2014). *ZFHX3* interacts with the protein inhibitor of activated STAT3 (PIAS3) (Liu et al., 2014; Tomomori et al., 2018), which regulates paracrine circuits. Increased expression of STAT3 contributes to cell proliferation, transformation, inflammation, angiogenesis, migration, matrix deposition, and eventual structural changes in AF (Nojiri et al., 2004; Park et al., 2013; Liu et al., 2014; Tomomori et al., 2018; Alí et al., 2019). Although the roles of *ZFHX3* in extra-PV triggers after AFCA have never been evaluated, we identified 5 SNPs of *ZFHX3* associated with extra-PV triggers, which are a significant predictor of clinical recurrence of AF after catheter ablation. Therefore, extra-PV triggers appear to be influenced by both acquired and genetic factors and are more commonly found in structurally remodeled AF (Gabbiani et al., 1978; Park et al., 2013; Alí et al., 2019). Although *ZFHX3* plays negative roles in structural remodeling and AF progression (Lubitz et al., 2014; Zaw et al., 2017), it can play a positive role in contributing to permanent conduction block by scar maturation at PV isolation lesion sites (Tomomori et al., 2018).

### Genetic Influence in Atrial Fibrillation Catheter Ablation

AF is a heritable disease, and more than 100 AF-associated genetic loci have been demonstrated through GWAS (Roselli et al., 2018). In studies observing the clinical outcome of AF according to the genetics, the top AF-associated gene *PITX2* affects the anti-arrhythmic drug response and response to electrical cardioversion (Parvez et al., 2012, 2013). Husser et al. (2010) and Shoemaker et al. (2013) reported that AF genetic loci had a significant effect on the rhythm outcome after AFCA in small studies. On the other hand, Choi et al. (2015) reported that AF-associated genetic loci did not affect the rhythm outcome after AFCA in a study of 1,068 AF patients. These discordant results might be due to the ethnic difference, possibility of proxy SNPs, and sample sizes (Choi et al., 2015). In this study, we showed that the second most highly AF associated gene, *ZFHX3*, had a significant relationship with extra-PV triggers but not with the rhythm outcome of AFCA.

### Limitations

This study had several limitations. First, it was a single-center, retrospective, observational cohort study that might have had a selection bias. The extra-PV trigger-associated SNPs were replicated internally. Second, we only included patients who underwent an isoproterenol provocation test and excluded those who did not undergo a provocation test or GWAS. Third, although we used a consistent isoproterenol provocation protocol, it was not standardized globally or reproducibly. Fourth, we tried to eliminate extra-PV triggers as much as we could during the procedure, but the actual trigger might have existed in the epicardial layer or deep inside the septum.

## Conclusion

The second most highly AF associated gene, *ZFHX3* and acquired LA remodeling were associated with extra-PV trigger. However, extra-PV triggers were independent predictor of AF recurrence after AFCA, the *ZFHX3* did not show genetic effects on AF recurrence.

## Data Availability Statement

The original contributions presented in the study are included in the article/Supplementary Material, further inquiries can be directed to the corresponding author/s.

## Ethics Statement

The studies involving human participants were reviewed and approved by the Institutional Review Board at Yonsei University Health System. The patients/participants provided their written informed consent to participate in this study.

## Author Contributions

IH contributed to data anylsis and wrote the manuscript. O-SK was involved in data anaylsis and interpretation. MH was participated in genetic anaylsis. S-YY was participated in data acquisition. J-WP contributed to clinical data acquisition and statistical interpretation. HY and T-HK contributed to clinical data acquisition and clinical interpretation. J-SU and BJ contributed to data acquisition, clinical, and statistical interpretation. M-HL was involved in overall clinical data acquisition and data review. H-NP contributed to the study design, study protocol design, data review, and wrote the manuscript. All authors contributed to the article and approved the submitted version.

## Conflict of Interest

The authors declare that the research was conducted in the absence of any commercial or financial relationships that could be construed as a potential conflict of interest.

## Publisher’s Note

All claims expressed in this article are solely those of the authors and do not necessarily represent those of their affiliated organizations, or those of the publisher, the editors and the reviewers. Any product that may be evaluated in this article, or claim that may be made by its manufacturer, is not guaranteed or endorsed by the publisher.
