# Corrigendum: Association of ZFHX3 genetic polymorphisms and extrapulmonary vein triggers in patients with atrial fibrillation who underwent catheter ablation

**DOI:** 10.3389/fphys.2022.1100597

**Published:** 2022-12-02

**Authors:** Inseok Hwang, Oh-Seok Kwon, Myunghee Hong, Song-Yi Yang, Je-Wook Park, Hee Tae Yu, Tae-Hoon Kim, Jae-Sun Uhm, Boyoung Joung, Moon-Hyoung Lee, Hui-Nam Pak

**Affiliations:** Yonsei University Health System, Seoul, South Korea

**Keywords:** atrial fibrillation, ZFHX3, genetic polymorphism, extra-pulmonary vein, recurrence, catheter ablation

In the published article, there were errors in the **Funding statement**. [H21C0011 and NRF-2020R1A2B01001695]. The correct Funding statement appears below.

“Funding

This work was supported by grants (HI19C0114 and HI21C0011) from the Ministry of Health and Welfare and by a grant (NRF-2020R1A2B5B01001695) from the Basic Science Research Program of the National Research Foundation of Korea (NRF), funded by the Ministry of Science, ICT, and Future Planning (MSIP).”

The authors apologize for this error and state that this does not change the scientific conclusions of the article in any way. The original article has been updated.

