# Supplementary material for: Association of ZFHX3 Genetic Polymorphisms and Extra-Pulmonary Vein Triggers in Patients With Atrial Fibrillation Who Underwent Catheter Ablation
Source: Front Physiol. 2022 Jan 5;12:807545. doi: 10.3389/fphys.2021.807545 (PMC8766666; doi:10.3389/fphys.2021.807545)
Supplement: Supplementary file 1 [file Data_Sheet_1.docx]

***Supplementary Material***

**Supplementary Table 1. Patient characteristics of the study groups**

|  | **Overall**  **(n=1782)** | **Cohort 1**  **(n=891)** | **Cohort 2**  **(n=891)** | ***p*** |
| --- | --- | --- | --- | --- |
| **Age, years** | 59.4±10.8 | 59.3±10.8 | 59.4±10.7 | 0.725 |
| **Gender (Male), n (%)** | 1309 (73.5%) | 657 (73.7%) | 652 (73.2%) | 0.789 |
| **PAF, n (%)** | 1169 (65.9%) | 579 (65.3%) | 590 (66.4%) | 0.628 |
| **AF duration, months** | 38.8±43.6 | 39.4±42.5 | 38.2±44.7 | 0.415 |
| **Comorbidities** |  |  |  |  |
| **Heart failure, n (%)** | 238 (13.4%) | 122 (13.7%) | 116 (13.0%) | 0.676 |
| **Hypertension, n (%)** | 803 (45.1%) | 404 (45.3%) | 399 (44.8%) | 0.812 |
| **DM, n (%)** | 258 (14.5%) | 134 (15.0%) | 124 (13.9%) | 0.501 |
| **Stroke, n (%)** | 197 (11.1%) | 106 (11.9%) | 91 (10.2%) | 0.257 |
| **Vascular disease, n (%)** | 190 (10.7%) | 86 (9.7%) | 104 (11.7%) | 0.167 |
| **BMI** | 24.9±3.0 | 24.9±2.9 | 24.9±3.0 | 0.626 |
| **CHA2DS2-VASc Score** | 1.7±1.5 | 1.8±1.5 | 1.7±1.5 | 0.780 |
| **Echocardiographic parameters** |  |  |  |  |
| **LA dimension** | 41.3±6.2 | 41.3±5.9 | 41.4±6.4 | 0.987 |
| **LA voltage** | 1.5±0.7 | 1.5±0.7 | 1.5±0.7 | 0.375 |
| **LV ejection fraction** | 63.4±8.1 | 63.3±8.0 | 63.6±8.2 | 0.368 |
| **E/Em** | 10.2±4.2 | 10.2±4.1 | 10.2±4.3 | 0.794 |
| **Clinical recurrence, n (%)** | 592 (33.2%) | 304 (34.1%) | 288 (32.3%) | 0.421 |

PAF: Paroxysmal atrial fibrillation, DM: Diabetes mellitus, BMI: Body mass index, TIA: Transient ischemic attack, LA: Left atrium, LV: Left ventricle

**Supplementary Table 2. Association between extra-PV triggers and the *ZFHX3* SNPs**

| **Extra-PV trigger** | **Cohort 1**  **(n=891)** | | |  | **Cohort 2**  **(n=891)** | | |
| --- | --- | --- | --- | --- | --- | --- | --- |
| **SNP** | **OR** | **CI (95%)** | ***p*** |  | **OR** | **CI (95%)** | ***p*** |
| rs13336412 | 0.74 | 0.55-1.00 | 0.049 |  | 0.64 | 0.48-0.86 | 0.003 |
| rs61208973 | 1.39 | 1.04-1.87 | 0.028 |  | 1.35 | 1.02-1.78 | 0.035 |
| rs2106259 | 1.46 | 1.08-1.97 | 0.014 |  | 1.37 | 1.02-1.84 | 0.034 |
| rs12927436 | 1.39 | 1.03-1.88 | 0.032 |  | 1.45 | 1.09-1.92 | 0.010 |
| rs1858801 | 1.38 | 1.02-1.87 | 0.037 |  | 1.52 | 1.15-2.00 | 0.003 |
| rs148371120 | 2.18 | 1.11-4.27 | 0.023 |  | NA | NA | NA |
| rs8055870 | 0.48 | 0.31-0.75 | 0.001 |  | NA | NA | NA |
| rs57263129 | 0.68 | 0.50-0.93 | 0.016 |  | NA | NA | NA |
| rs61164185 | 0.72 | 0.54-0.97 | 0.031 |  | NA | NA | NA |
| rs117315995 | NA | NA | NA |  | 0.457 | 0.26-0.79 | 0.005 |
| rs72793298 | NA | NA | NA |  | 1.507 | 1.01-2.25 | 0.045 |
| rs7500397 | NA | NA | NA |  | 1.328 | 1.00-1.75 | 0.046 |
| rs58670562 | NA | NA | NA |  | 0.711 | 0.52-0.96 | 0.028 |
| rs7199343 | NA | NA | NA |  | 0.429 | 0.19-0.98 | 0.046 |

SNP: Single nucleotide polymorphism, OR: Odds ratio, CI: Confidence interval

**Supplementary Table 3. Extra-PV trigger regions based on the SNPs**

| **Extra-PV trigger regions** | **rs13336412 (+)**  **(n=140)** | **rs13336412 (-)**  **(n=38)** | ***p*** | **rs61208973 (+)**  **(n=140)** | **rs61208973 (-)**  **(n=38)** | ***p*** |
| --- | --- | --- | --- | --- | --- | --- |
| BB+Septum | 37 (21.6%) | 6 (22.2%) | 0.945 | 32 (22.9%) | 11 (28.9%) | 0.437 |
| CS+Mitra annulus | 27 (15.8%) | 3 (11.1%) | 0.773 | 24 (17.1%) | 6 (15.8%) | 0.843 |
| HRA+LRA+SV | 18 (10.5%) | 0 (0.0%) | 0.140 | 140 (100.0%) | 38 (100.0%) | NA |
| LAA and LOM | 9 (5.3%) | 2 (7.4%) | 0.649 | 8 (5.7%) | 3 (7.9%) | 0.704 |
| LAPW | 8 (4.7%) | 1 (3.7%) | 1.000 | 6 (4.3%) | 3 (7.9%) | 0.404 |
| Multiple | 40 (23.4%) | 6 (22.2%) | 0.894 | 38 (27.1%) | 7 (18.4%) | 0.273 |
| Others | 10 (5.8%) | 1 (3.7%) | 1.000 | 10 (7.1%) | 0 (0.0%) | 0.122 |
| SVC | 22 (12.9%) | 8 (29.6%) | 0.039 | 22 (15.7%) | 8 (21.1%) | 0.436 |
| RA | 40 (23.4%) | 8 (29.6%) | 0.482 | 22 (15.7%) | 8 (21.1%) | 0.436 |
| LA | 44 (25.7%) | 6 (22.2%) | 0.697 | 38 (27.1%) | 12 (31.6%) | 0.589 |
| Septum | 37 (21.6%) | 6 (22.2%) | 0.945 | 32 (22.9%) | 11 (28.9%) | 0.437 |

BB: Bachmann’s bundle, CS: Coronary sinus, HRA: High right atrium, LRA: Low right atrium, SV: Superior venarum, LAA: Left atrial appendage, LOM: Ligament of Marshall, LAPW: Left atrial posterior wall, SVC: Superior vena cava

RA included HRA, LRA, SV and SVC. LA included CS, mitral annulus, LAA+LOM, and LAPW. Septum included septum, and BB.

**Supplementary Figure legend**

**Supplementary Figure 1. AF recurrence according to the *ZFHX3* genotype**

The AF recurrence rate was higher in the extra-PV trigger (+) *ZFHX3* (+) group **(A)**, and the AF recurrence rate did not differ regardless of the *ZFHX3* genotype in the extra-PV trigger (-) group **(B)**
