# Supplementary figures and images for: Association of ZFHX3 Genetic Polymorphisms and Extra-Pulmonary Vein Triggers in Patients With Atrial Fibrillation Who Underwent Catheter Ablation
Source: Front Physiol. 2022 Jan 5;12:807545. doi: 10.3389/fphys.2021.807545 (PMC8766666; doi:10.3389/fphys.2021.807545)

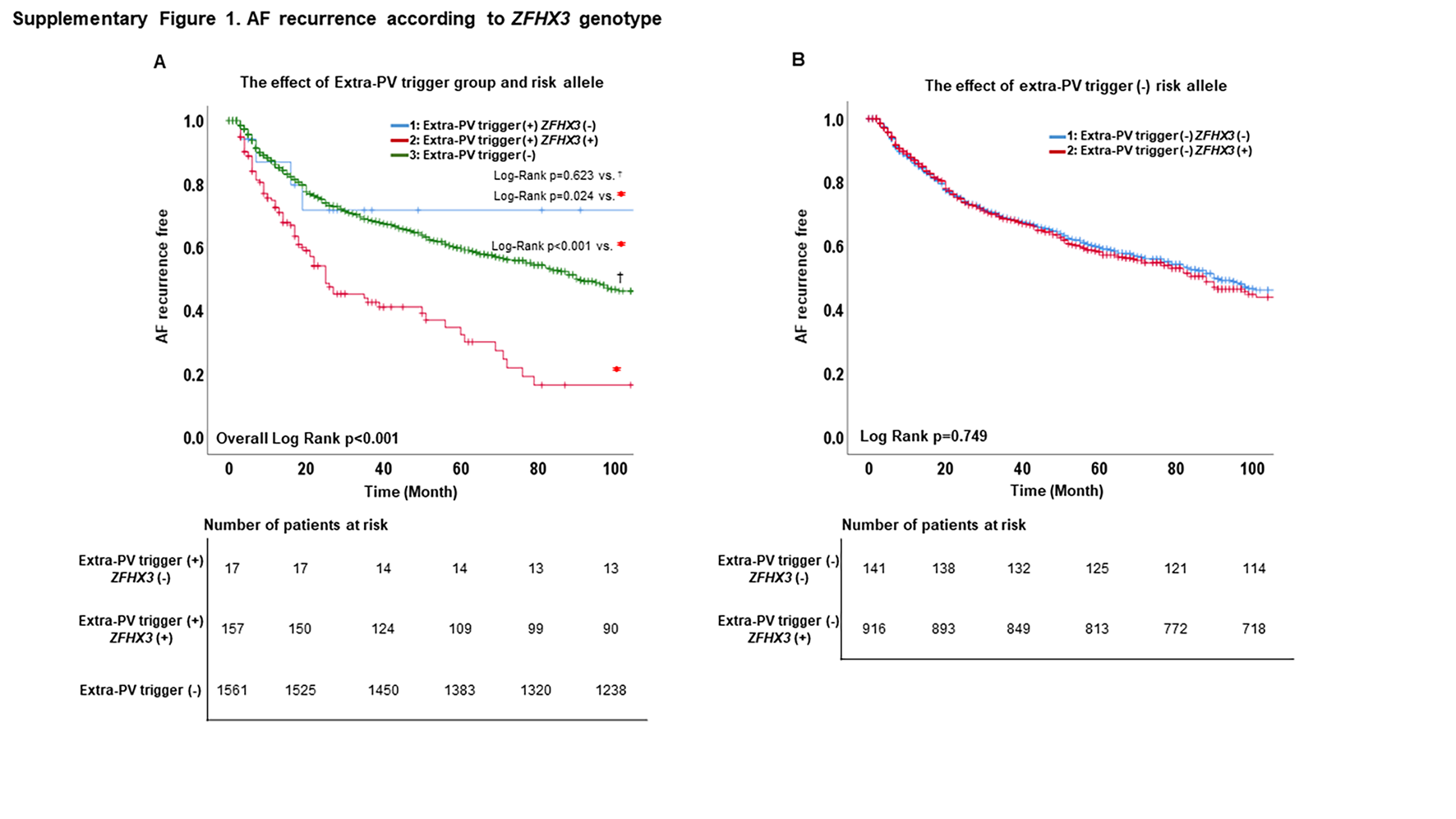

Supplement: Supplementary file 2 [file Image_1.TIF]
